# Atrophy of the Posterior Subiculum Is Associated with Memory Impairment, Tau- and Aβ Pathology in Non-demented Individuals

**DOI:** 10.3389/fnagi.2017.00306

**Published:** 2017-09-20

**Authors:** Olof Lindberg, Gustav Mårtensson, Erik Stomrud, Sebastian Palmqvist, Lars-Olof Wahlund, Eric Westman, Oskar Hansson

**Affiliations:** ^1^Clinical Memory Research Unit, Department of Clinical Sciences, Lund University Lund, Sweden; ^2^Division of Clinical Geriatrics, Department of Neurobiology, Care Sciences and Society, Karolinska Institutet Stockholm, Sweden

**Keywords:** hippocampus subfield, beta amyloid, subjective cognitive decline, mild cognitive impairment, preclinical AD

## Abstract

Alzheimer’s disease (AD) is associated with atrophy of the cornu ammonis (CA) 1 and the subiculum subfield of the hippocampus (HC), and with deficits in episodic memory and spatial orientation. These deficits are mainly associated with the functionality of the posterior HC. We therefore hypothesized that key AD pathologies, i.e., β-amyloid and tau pathology would be particularly associated with the volume of the posterior subiculum in non-demented individuals. In our study we included 302 cognitively normal elderly participants (CN), 183 patients with subjective cognitive decline (SCD) and 171 patients with amnestic mild cognitive impairment (MCI), all of whom underwent 3T magnetic resonance images (MRI). The subicular subfield was segmented using Freesurfer 5.3 and divided into 10 volumetric segments moving from the most posterior (segment 1) to the most anterior part along the axis of the hippocampal head and body (segment 10). Cerebrospinal fluid (CSF) Aβ_42_ and phosphorylated tau (P-tau) were quantified using ELISA and were used as biomarkers for β-amyloid and tau pathology, respectively. In the total sample, tau-pathology and Aβ-pathology and (measured by elevated P-tau and low Aβ_42_ levels in CSF) and mild memory dysfunction were mostly associated with the volume changes of the posterior subiculum. Both SCD and MCI patients with elevated P-tau or low Aβ_42_ levels displayed predominantly posterior subicular atrophy in comparisons to control subjects with normal CSF biomarker levels. Finally, there was no main effect of Aβ_42_ or P-tau when comparing SCD with abnormal P-tau or Aβ_42_ with SCD with normal levels of these CSF-biomarkers. However, in the left subiculum there was a significant interaction revealing atrophy in the left posterior but not the anterior subiculum in participants with low Aβ_42_ levels. The same pattern was observed on the contralateral side in participants with elevated P-tau levels. In conclusion, AD pathologies and mild memory dysfunction are mainly associated with atrophy of the posterior parts of the subicular subfields of the HC in non-demented individuals. In light of these findings we suggest that segmentation of the HC subfields may benefit from considering the volume of the different anterior-posterior subsections of each subfield.

## Introduction

Atrophy of the hippocampus (HC) is common in many diseases affecting the brain, such as Alzheimer’s disease (AD; Braak and Braak, 1991), frontotemporal lobar degeneration (FTLD; Lindberg et al., 2012), schizophrenia (Bogerts et al., 1993; Altshuler et al., 1998; Heckers, 2001), depression (Bremner et al., 2000; Vakili et al., 2000; Campbell et al., 2004) and post-traumatic stress disorder (Van Rooij et al., 2015). The HC can from a gross anatomical perspective be divided into three sub-parts moving from anterior to posterior: the hippocampal head, the hippocampal body and the hippocampal tail; further described in Section “A Gross Anatomical Division of the Hippocampus”) and in Malykhin et al. (2007).

Further, on the basis of the characteristics of the cellular cytoarchitecture, the HC can be divided into six different subfields: The cornu ammonis (CA) 1–4, the dentate gyrus and the subiculum (Duvernoy and Bourgouin, 1998). Various subfields may be implicated in different forms of brain dysfunction. For example, AD is in early stages associated with atrophy of primarily CA1 and subiculum (Carlesimo et al., 2015; for review see de Flores et al., 2015). CA1 is also vulnerable to ischemia and mesial lobe epilepsy, CA2 to schizophrenia and subiculum to aging and several other forms of dementia such as FTLD (Van Hoesen and Hyman, 1990; Tabuchi et al., 1992; West et al., 1994; Bobinski et al., 1998; Adriano et al., 2012; Lindberg et al., 2012). Since different risk factors and different diseases have partly unique patterns of cellular degeneration, several parcellation protocols have been developed to obtain the volume of each subfield from structural magnetic resonance images (MRI; reviewed in Yushkevich et al., 2015). What is in common for most of these protocols is that they involve segmentation at very high resolution perpendicular to the long axis of HC. One single coronal slice of HC may contain all six subfields. In contrast, some subfields such as the subiculum are present on all slices along the long axis of the HC. The boundary between the subiculum and other subfields in the coronal view is often defined at sub-voxel level but spans several centimeters on the long axis of the HC.

While this is a valid division from a neuropathological perspective (Duvernoy and Bourgouin, 1998), it is less relevant from a functional perspective. The anterior CA1 and subiculum project to the amygdala, the prefrontal cortex, the temporal pole and the nucleus accumbens (Fanselow and Dong, 2010). The posterior parts of these regions project to the mammillary bodies, the retrosplenial cortex and the anterior cingulate (Aggleton, 2012). The anterior and posterior CA1 and subiculum are in fact involved in such different functions that some authors suggests that the dorsal (posterior in primates) and ventral (anterior in primates) parts of the HC could potentially be regarded as two functionally distinct structures (Fanselow and Dong, 2010). The posterior HC supports functions of locomotion, orientation, movement, navigation and exploration (Fanselow and Dong, 2010) and in detailed spatial and autobiographical (episodic) memory (Strange et al., 2014), while the anterior HC is associated with motivational behavior, neuroendocrine and autonomic (hypothalamic) functions (Fanselow and Dong, 2010). In view of the clinical symptoms of AD with deficits of episodic memory and spatial orientation, it could be hypothesized that AD-pathology may selectively affect the posterior HC.

One approach to investigate differences along the axis of the HC is to divide the HC or an HC subfield on the basis of gross anatomical landmarks of the hippocampal head, body and tail. Using this approach, one study found that both AD and semantic dementia were associated with atrophy of the CA1 and subiculum. However, these subfields were more atrophic in the hippocampal head in the semantic dementia group. The same study also investigated the effect of Aβ pathology in patients with mild cognitive impairment (MCI). MCI patients with Aβ pathology displayed atrophy in the CA1 and the subiculum, while MCI patients without Aβ pathology only had atrophy in the CA1 (La Joie et al., 2013).

Another way to investigate how atrophy is distributed along the anterior-posterior axis of HC is to perform shape analysis of the whole HC. While this approach often shows involvement of the hippocampal body in AD (Gerardin et al., 2009; Lindberg et al., 2012; Tang et al., 2016), the method is limited in the sense that it does not produce volumetric data on specific subfields.

The purpose of this study is to further develop the hippocampal subfields segmentation of the presubiculum (defined as the subiculum in this study) from Freesurfer (FS) 5.3. We aim to study volumetric differences along the anterior-posterior axis of this region in relation to Aβ_42_ and P-tau levels in cerebrospinal fluid (CSF; as a proxy for tau and Aβ pathology) and memory performance in cognitively normal participants (CN), subjects with subjective cognitive decline (SCD) and subjects with amnestic MCI.

## Materials and Methods

### Study Participants

All subjects gave written consent to participate in the study. Ethical approval for the study was given by the Regional Ethical Review Board in Lund. The study population was recruited from the Swedish BioFINDER study (Biomarkers for Identifying Neurodegenerative Disorders Early and Reliably)^1^. Cognitively normal and non-demented patients with mild cognitive symptoms characterized as having SCD or MCI were included. CN subject were originally enrolled from the population-based cohort (Manjer et al., 2001; Riboli, 2001; Mattsson et al., 2016). The inclusion criteria were: age ≥60 years old, MMSE 27–30, a Clinical Dementia Rating scale score of 0 and fluent in Swedish. Exclusion criteria were: presence of SCD, MCI or dementia, significant neurologic disease (including stroke, Parkinson’s disease and multiple sclerosis), severe psychiatric disease (including severe depression or psychotic syndromes), and refusing lumbar puncture or MRI. All CN subjects underwent a thorough clinical assessment including neurological, psychiatric and cognitive testing, all performed by a medical doctor, in addition to MRI of the brain and relevant blood and CSF sampling.

The SCD and MCI cases were recruited consecutively and were thoroughly assessed by physicians with special competence in dementia disorders. The inclusion criteria were: referred to a memory clinic due to possible cognitive impairment, not fulfilling the criteria for dementia, MMSE 24–30, age 60–80 years and, fluent in Swedish. The exclusion criteria were: cognitive impairment that without doubt could be explained by another condition (other than prodromal dementia), severe somatic disease and refusing lumbar puncture or neuropsychological investigation. The classification in SCD or MCI was based on a neuropsychological battery and the clinical assessment of a senior neuropsychologist together with two physicians experienced in dementia disorders (OH and SP). The neuropsychological battery included tests for verbal ability (multiple-choice vocabulary tests and semantic verbal fluency), episodic memory (Rey Auditory Verbal Learning Test and Rey Complex Figure Test—delayed recall), visuospatial ability (Block Design and the copy trial of Rey Complex Figure Test), attention and executive functions (Trail Making Test and Letter Verbal Fluency). SCD was defined as being referred to a memory clinic due to cognitive complaints but not showing signs of objective cognitive impairment in the neuropsychological battery. MCI was defined as having objective cognitive impairment on the neuropsychological battery in agreement with the consensus criteria for MCI described (Petersen, 2004).

### CSF Analyses

The procedure and analysis of the CSF followed the Alzheimer’s Association Flow Chart for CSF biomarkers (Blennow et al., 2010). Lumbar CSF samples were analyzed at the same time according to a standardized protocol (Palmqvist et al., 2014). CSF total tau (T-tau), and Aβ_42_ were analyzed by EUROIMMUN (^EI^) enzyme-linked immunosorbent assays (ELISAs; EUROIMMUN AG, Lübeck, Germany). Tau phosphorylated at Thr181 (P-tau) were analyzed with INNOTEST (^IT^) ELISAs (Fujirebio Europe, Ghent, Belgium).

Since the levels of CSF Aβ_42_ are bimodally distributed, we dichotomized the levels into normal and elevated levels. An unbiased cutoff value of Aβ_42_ levels (≤527 nanogram/liter; ng/l) was established using mixture modeling and the R (v. 3.0.1, The R Foundation for Statistical Computing), as described in previous publication (Palmqvist et al., 2014). CSF P-tau was treated as a continuous variable when correlations with subicular volume segments were performed. Furthermore, the P-tau was dichotomized using a previously published cut-off low ≤52 and high >52 ng/l (Mulder et al., 2010). In this cohort, a cut-off of 52 ng/l was shown to selectively affect the posterior subiculum, while a higher cut-off (70 ng/l) affected both the anterior and posterior subiculum Supplementary Figure S1.

### Assessment of Memory Function

Memory function was measured using the delayed recall task from the AD Assessment Scale—cognition (ADAS-cog; Rosen et al., 1984). The test was administered by first having the participant read 10 words and then immediately recall them. This was repeated three times to ensure a sufficient encoding process. After a distraction task (ADAS-cog Naming Objects and Fingers) the participant was asked to freely recall as many of the 10 words as possible. The score was recorded as the number of errors/forgotten words.

### MRI Acquisition

T1-weighted images were obtained on a single 3 tesla MR scanner (Trio, Siemens, Germany). Volumetric analysis was performed on T1-weighted 3D MP-RAGE image (TR = 1950 ms TE = 3, 4 ms) with 1 mm isotropic voxels and 178 slices. Hippocampal subfield segmentation was performed using FS image analysis pipeline version 5.3.0, which is documented and freely available for download online^2^. All image processing was performed within TheHiveDB database (Muehlboeck et al., 2014). The subfield parcellation method implemented in FS 5.3 has been criticized, particularly for the definition of the subfields in the most anterior part of the HC (Wisse et al., 2014).

**Figure 1:**
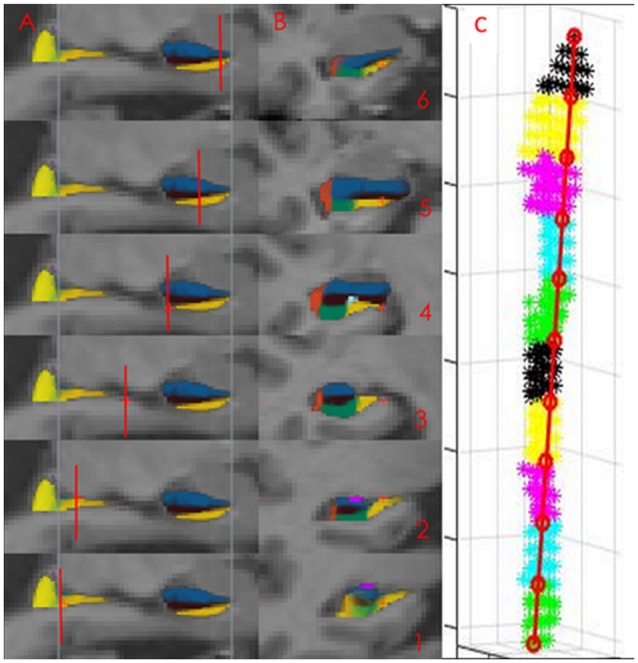
The presubiculum subfield in Freesurfer (FS) 5.3. The Panels (**A**, sagittal, **B**, coronal) displays the presubicular (denoted subiculum in this study) subfield (yellow color) segmented in FS 5.3. Images 1–3 display the hippocampal body, images 4–6 the hippocampal head. The subfield ends in the most posterior part of the hippocampal body (displayed as the most posterior blue line in sagittal view and in the most anterior slice of the hippocampal head displayed by the most anterior blue line in sagittal view). Short red lines illustrate the position of the coronal slices. At coronal view image (1) is only the medial yellow label part of the presubicular subfield. The lateral part is the hippocampal tail. Panel **(C)** displays the parcellation of presubiculum into 10 volumetric segments in one individual.

While we agree with this criticism, we suggests that the subfield defined as presubiculum in FS 5.3 always measures the volume of a part or even the whole subiculum as defined in other protocols (for comparison on other protocols definition of subiculum see Yushkevich et al., 2015) and Figures 1A,B. Further, a detailed comparison between the segmentation procedure implemented in FS 5.3 and neuropathological data indicates that most of the part labeled presubiculum in FS 5.3 is a part of the subiculum (compare Figures 1A,B with results in Ding and Van Hoesen, 2015; or the definition of subiculum in a quantitative neuropathological study, Amunts et al., 2005). Moreover, the presubiculum in FS 5.3 never invades gray matter that belongs to another subfield, which has been pointed out to be another problem with the segmentation method used in FS 5.3 (Wisse et al., 2014). During visual inspection we further found that the segmentation of the presubiculum was generally of excellent quality. The reason for this is that this subfield is located adjacent to white matter inferiorly and to the hippocampal fissure superiorly. White matter and the fissure have different signal intensity than gray matter which makes it easy to identify the anterior and inferior boundary of the subfield.

Finally have a high-resolution functional imaging (fMRI) study found that the changes in connectivity between the anterior-posterior HC are greatest in the subiculum (Libby et al., 2012). The definition of the subiculum in this fMRI study almost completely overlaps with the definition of the presubiculum in FS. 5.3 (compare Figure 1, with Figure 1 in Libby et al., 2012).

### Dividing the Subicular Subfield into 10 Volumetric Segments from Anterior to Posterior

The segmentation of the subicular subfield is represented as a probability map in FS with intensity 0 (not belonging to the subfield) to 255 (belonging to the subfield with 100% certainty). The specified subfields of the HC were binarized at the threshold of 127 and extracted using Freesurfer 5.3. This threshold value corresponded to a probability of 0.5 or higher that the voxel belonged to the specified subfield. The masks were imported into MATLAB 2015b (Natick, MA, USA; The MathWorks, Inc) where the two voxels on the surface with the greatest Euclidian distance between them were identified. A straight line was drawn between these two voxels, from the most posterior to the most anterior voxel. This line was divided into 10 equally spaced segments. Each voxel was projected onto this line and was assigned to the segment into which it was projected. The volume of each segment was calculated as the number of voxels assigned to that segment multiplied by the voxel volume (Figure 1C).

Visual inspection was not possible for each partition along the anterior-posterior axis (as it was not possible to visually identify the boundary of each partition). To further ensure the quality of the data a volume of an individual partition that was more than plus or minus three standard deviations from the mean value was excluded from analysis. This threshold corresponded to the variation found in the total volume of the left subiculum in CN subjects. The variation of a single partition was thus not allowed to deviate from the mean more than that which could be found for the total volume of the left subiculum. Very few measurements (0.84% of 6560 measurements) exceeded 3 SD from the mean.

### A Gross Anatomical Division of the Hippocampus

Each subicular volume segment encompasses 10% of the total distance from the most anterior part of the hippocampal head to the most posterior part of the hippocampal body. Since subfield segmentation of the hippocampal tail is not available in FS 5.3 or in FS 6 this part could not be included in the analysis. The most posterior slices of the hippocampal head can be defined as the first slice where the uncal apex is clearly presented. The most anterior part of the hippocampal tail can be defined as the first slice where the fornix is clearly seen in full profile or is separated from the wall of the ventricle (Duvernoy and Bourgouin, 1998; Malykhin et al., 2007). On the MNI template (the standard brains from the Montreal Neurological Institute), the hippocampal head encompasses approximately 47% of the total length of the hippocampal head + the hippocampal body (total HC) and the hippocampal body, approximately 53%. Thus, subicular volume segments 1–5 are located in the body of the HC, while transition between the hippocampal body and the hippocampal head occur in segment 6.

### Statistical Analysis

The statistical analysis was performed using Statistica 12 (Tulsa, USA) and SPSS (Armonk, NY, USA: IBM Corp). A general linear model (GLM) with status of biomarker (high vs. low Aβ_42_ or P-tau) or number of forgotten items (0–1, 2–3, 4–5 and 6–10 items) and gender entered as factors and age and intracranial volume as covariates was used to investigate differences in the repeated measurements of the 10 volume segment of the subiculum. The presence or absence of one or two allele four in the apolipoprotein (*APOE*-status) did not significantly contribute to explaining the volume of the subiculum. This factor was therefore excluded from the model. For the correlation of P-tau in the total sample we used partial correlation including age and intracranial volume (ICV) as covariates in the model. A *p*-value <0.050 was considered significant in all models.

## Results

### All Participants

In total, 656 participants were included in the study. Demographic data is presented in Table 1. The total volume of left and right subiculum was smaller in MCI compared to CN and SCD. No difference was found between SCD and CN. In the total sample there was no volumetric difference between women and men on either side of the structure but the volume was bilaterally significantly correlated with age (both sides approximately *r* = −0.34).

**Table 1:** Demographical data.

	ALL	CN	SCD	MCI
*n*	656	302	183	171
F/M	352 (306)	182/120	100/83	102/69
Age	72.2 (5.5)	73.7 (5.0)	70.5 (5.7)*	71.3 (5.3)*
MMSE	28.4 (1.6)	29 (0.9)	28.5 (1.4)*	27 (1.8)**
Memory score, errors	3.6 (2.8)	2 (2)	3.4 (2)*	7 (2)**
CSF Aβ42	618 (216)	667 (192)	630 (222)	519 (218)**
CSF P-tau	58 (24)	54 (19)	57 (25)	67 (29)**
Left HC	3753 (612)	3884 (520)	3826 (595)	3441 (674)**
Right HC	3815 (604)	3929 (548)	3893 (579)	3546 (647)**
Aβ_42_ −/+	398/258	220/82	114/69	64/107
P-tau−/+	324/332	160/142	101/82	63/108
*APOE4*	401/252	216/85	108/73	77/94

Abbreviations: n, number; F/M, number female/male; MMSE, Mini Mental State Examination; Memory, Errors on 10-word list delayed recollection test; Aβ_42_, beta-amyloid 42 CSF-levels; P-tau, phosphorylated tau CSF-levels; HC, hippocampus; Aβ_42_*−/+*, Number amyloid negative/amyloid positive; P-tau−/+, number with normal/elevated levels of P-tau; *APOE4*, number without/with the *allele* 4; *sign diff from control; **sign diff from SCD and CTL (*p* < 0.050).

#### Relation to Aβ Pathology

**Figure 2:**
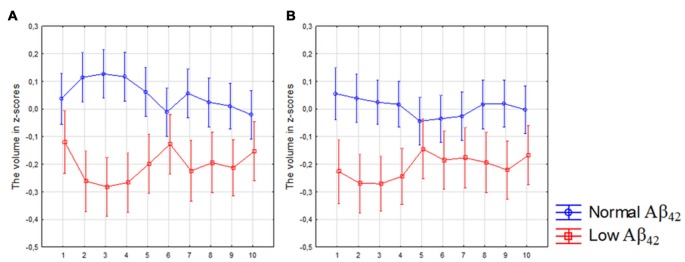
Differences in subicular volume between cases with normal and low cerebrospinal fluid (CSF) Aβ_42_ levels. The figure denotes differences in subicular volume segment in participants with low (red line) and normal (blue line) levels of Aβ_42_ in all participants. The *x*-axis denotes volume segment from posterior (segment 1) to anterior (segment 10). The *y*-axis denotes the volume of segment normalized as z-scores. **(A)** left side, **(B)** right side. Analysis is controlled for gender, intracranial volume and age. Vertical bars denote 95% confidence interval.

Participants with low CSF Aβ_42_ levels (*n* = 258) displayed volume loss of the left segments 1–5 and 7–9 and right subicular segments 1–4 and 8–9 compared with participants with normal levels. However, the volume loss was more significant in the posterior segments when compared to participants with normal CSF Aβ_42_ levels (Figures 2A,B). The analysis revealed a significant interaction between Aβ status (low ≤ 527 vs. high > 527 ng/l) and location (segments 1–10) in the left (*F*_(9,5598)_ = 3.40, *p* < 0.010), but not in the right subiculum.

#### Relation to Tau Pathology

**Figure 3:**
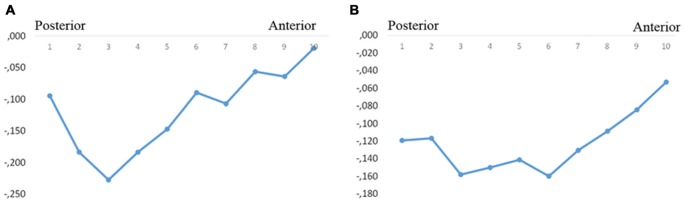
Correlations between subicular volume and CSF-P-tau levels. The figure denotes the correlation coefficients between the subicular volume segments and CSF P-tau in the whole group (**A**, left, **B**, right). *Y*-axis denotes the correlation coefficients. Dots denote volume segment moving from posterior (left) to anterior (right).

In the whole sample, the total volumes of left and right subiculum were significantly correlated with the levels of CSF P-tau treated as a continuous variable (both sides approximately *r* = −0.18; *p* < 0.010). The strongest correlation was found in the left segments 2–4 and right segments 1–6 (Figures 3A,B, Table 2).

**Table 2:** The correlation coefficients for the correlation between P-tau/delayed recall and subicular volume segment in all participants.

Vol	1	2	3	4	5	6	7	8	9	10
P-tau *r* Right	−0.12	−0.12	−0.16	−0.15	−0.14	−0.16	−0.13	−0.11	−0.08	−0.05
*p*	<0.010	<0.010	<0.010	<0.010	<0.010	<0.010	<0.010	0.010	0.032	0.181
P-tau *r* Left	−0.10	−0.18	−0.23	−0.18	−0.15	−0.09	−0.11	−0.06	−0.06	−0.02
*p*	0.021	<0.010	<0.010	<0.010	<0.010	0.021	0.010	0.150	0.102	0.630

*Abbreviations: Vol, volume segment; P-tau, phosphor tau; *r* Pearson’s correlation coefficient in partial correlation analysis including age and intracranial volume (ICV) as covariates; p, p value*.

#### Relation to Memory Function

The number of errors on *ADAS-cog delayed recall of a 10-word list* were significantly correlated with the total volume of the left (*r* = −0.41; *p* < 0.010) and the right (*r* = −0.36; *p* < 0.010) subiculum.

**Figure 4:**
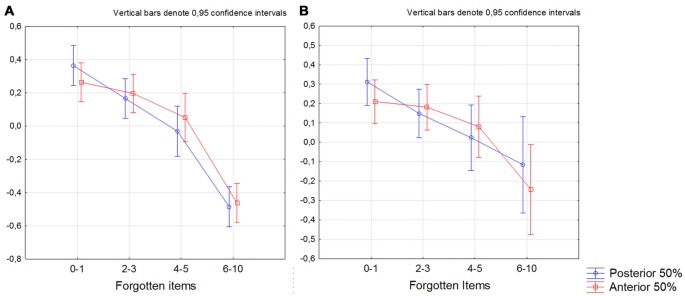
Differences between participants that forgot 0–1. 2–3. 4–5 and 6–10 items in the bilateral anterior and posterior 50% of the subiculum. The summarized volume of left+right 50% of posterior (blue line) and anterior (red line). *X*-axis denotes number of forgotten items. The *y*-axis denotes the volume of the anterior and the posterior 50% of the subiculum. **(A)** All participants, **(B)** participants with no cognitive decline on neuropsychological test (subjective cognitive decline (SCD)+cognitively normal (CN)). The model is further including gender as factor and age and intracranial volume (ICV) as covariates.

When dividing the sample into participants that forgot 0–1, 2–3, 4–5 and 6–10 items, we found that the bilateral posterior 50% of the subiculum was significantly smaller in participants that forgot 4–5 and 6–10 items compared to participants that forgot 0–1 items (*p* < 0.050 on Bonferroni *post hoc* test). For the anterior 50% of the subiculum we only found difference between participants that forgot 0–1 and 6–10 items (Figure 4A). The same results were obtained when only CN and SCD participants were included in the analysis (Figure 4B; Fischer’s* post hoc* (*p* < 0.050).

### SCD and MCI Analyzed Separately

#### SCD with Low CSF Aβ_42_ or Elevated CSF P-Tau Levels

**Figure 5:**
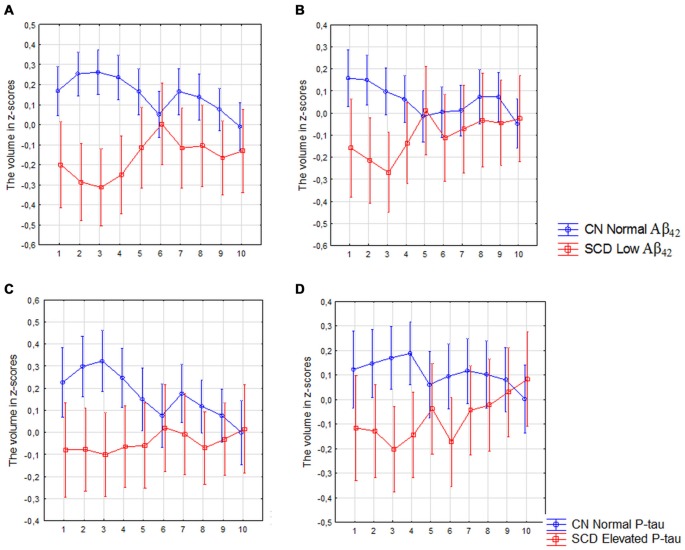
SCD with low Aβ_42_ or elevated P-tau CSF levels compared with controls with normal Aβ_42_ or P-tau levels. The figure denotes differences in subicular volume segment in SCD with abnormal (red line) and CN with normal (blue line) CSF biomarkers. **(A,B)** Display the volume segment in left **(A)** and right **(B)** subiculum in CN without and SCD participants with low CSF Aβ_42_. **(C,D)** Display the volume segment in left **(C)** and right **(D)** subiculum in CN without and SCD participants with elevated CSF P-tau. The *x*-axis denotes volume segment from posterior (segment 1) to anterior (segment 10). The *y*-axis denotes the volume of segment normalized as z-scores. The analysis is controlled for gender, intracranial volume and age. Vertical bars denote 95% confidence interval.

The analysis revealed a significant interaction between diagnosis (CN with normal CSF Aβ_42_ levels vs. SCD with low Aβ_42_ levels) and location (segments 1–10) in the left (*F*_(9,2430)_ = 3.00, *p* < 0.010) and right subiculum (*F*_(9,2430)_ = 2.00, *p* = 0.044; Figures 5A,B) as well as between diagnosis (CN with normal P-tau vs. SCD with elevated P-tau) and location in left subiculum (*F*_(9,2034)_ = 2.01, *p* = 0.027; Figure 5C). On the right side was this interaction however borderline significant (*F*_(9,2007)_ = 1.90, *p* = 0.045; Figure 5D).

#### MCI Patients with Low CSF Aβ_42_ or Elevated CSF P-Tau Levels

**Figure 6:**
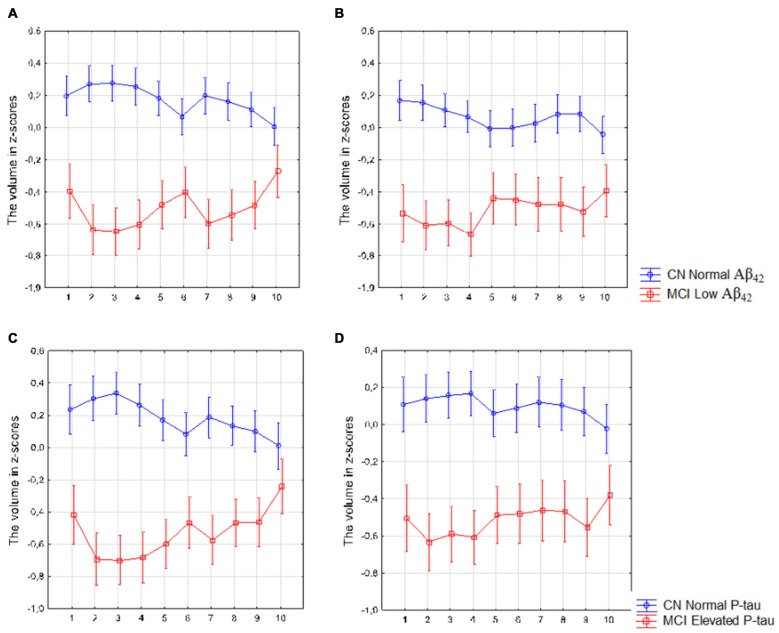
Mild cognitive impairment (MCI) with low Aβ_42_ or elevated P-tau levels CSF compared with controls with normal Aβ_42_ or P-tau levels. The figure denotes differences in subicular volume segment in MCI with abnormal (red line) and CN with normal (blue line) CSF biomarkers. **(A,B)** display the volume segment in left **(A)** and right **(B)** subiculum in CN without and MCI participants with low CSF Aβ_42_. **(C,D)** display the volume segment in left **(C)** and right **(D)** subiculum in CN without and MCI with elevated CSF P-tau. The *x*-axis denotes volume segment from posterior (segment 1) to anterior (segment 10). The *y*-axis denotes the volume of segment normalized as z-scores. Analysis is controlled for gender, intracranial volume and age. Vertical bars denote 95% confidence interval.

The analysis revealed a significant interaction between diagnosis (CN with normal Aβ_42_ vs. MCI with low Aβ_42_ levels) and location (segments 1–10) in the left (*F*_(9,2790)_ = 5.73, *p* < 0.010) and right (*F*_(9,2736)_ = 2.76, *p* < 0.010) subiculum (Figures 6A,B) as well as between diagnosis (CN with normal P-tau vs. MCI with elevated P-tau levels) and location in the left (*F*_(9,2268)_ = 7.18, *p* < 0.010) and right (*F*_(9,2196)_ = 1.96, *p* = 0.040) subiculum (Figures 6C,D).

#### SCD Patients with Low CSF Aβ_42_ or Elevated CSF P-Tau Levels Compared with SCD with Normal CSF Aβ_42_ or Elevated CSF P-Tau

**Figure 7:**
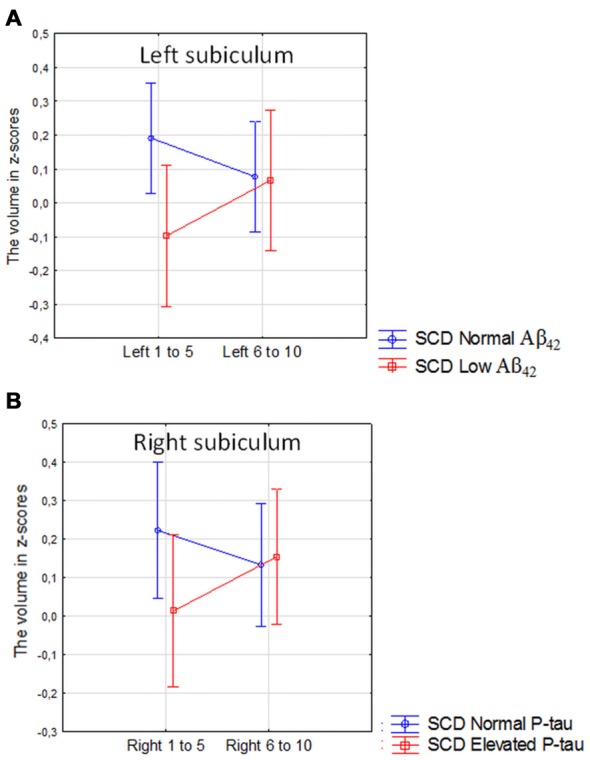
SCD with normal vs. SCD with low CSF Aβ_42_ or elevated P-tau. The figure denotes the interaction between high vs. low levels of CSF Aβ_42_ and the anterior vs. posterior 50% of the left subicular subfield **(A)**, and the interaction between high vs. low P-tau levels and the anterior vs. posterior 50% of the right subicular subfield **(B)**. Blue line, normal CSF levels, red line, abnormal CSF levels. Vertical bars denote 95% confidence interval.

There was no main effect of CSF Aβ_42_ or elevated CSF P-tau in the comparison between high vs. low CSF biomarkers in the SCD group. However, there was a borderline significant interaction between low vs. high levels of CSF Aβ_42_ and location (the anterior 50% vs. posterior 50% of the subicular subfield) in the left subiculum (Figure 7A; Current effect: *F*_(1,176)_ = 3.93, *p* = 0.049). For high vs. low P-tau levels the same effect was seen on the contralateral side (Figure 7B, Current effect: *F*_(1,179)_ = 4.34, *p* = 0.041).

## Discussion

The purpose of this study was to investigate whether a consideration of differences between anterior and posterior parts of a hippocampal subfield could improve our knowledge about how different disease pathologies affect the region. A suitable model to test this hypothesize is early AD. There is evidence that early clinical symptoms of AD, such as deficits in episodic memory (Ranganath and Ritchey, 2012) and scene processing (Lee et al., 2008; Ranganath and Ritchey, 2012; Strange et al., 2014), are associated with the functionality of the posterior hippocampal system. Further, it has been shown that the subiculum is commonly affected in AD.

In accordance with our hypothesis we found that Aβ pathology (measured with CSF Aβ_42_) and tau pathology (measured with CSF P-tau) were more associated with the volumes of the posterior parts than with the anterior parts of the subiculum. Further, we found that in SCD patients, low CSF Aβ_42_ levels were associated with reduced volume of left posterior but not anterior subiculum in comparison with SCD subjects with normal levels of Aβ_42_. The same effect was found with regard to tau pathology on the contralateral side. Interestingly we found a significant interaction between diagnosis and location (anterior vs. posterior atrophy) in both SCD and MCI with low Aβ_42_ or elevated P-tau levels when comparing these participants with CN with normal levels of the CSF biomarkers. Exploring this interaction further revealed that the patient group had more pronounced atrophy in posterior than the anterior subiculum. Such interaction was not present in SCD with normal levels of Aβ_42_ or P-tau, and modest in MCI with normal levels of Aβ_42_ (Supplementary Figures S2, S3). The results found in the SCD and MCI subjects could potentially be interpreted in terms of progression. The largest difference between anterior and posterior segments is seen in SCD subjects with abnormal CSF biomarkers (Figure 7). In MCI with abnormal CSF biomarkers the interaction effect is still significant. When inspecting analysis in Figure 6 we can however see that the whole left subiculum is significantly atrophied in MCI with low CSF Aβ_42_-levels, which could indicate that these patients have progressed so far in the disease process that atrophy has spread also to the anterior part of the subiculum.

In the total sample, we found that P-tau levels >52 ng/l were associated with decreased volume of the left posterior but not anterior subiculum in participants with normal Aβ_42_ levels. Both the anterior and the posterior left subiculum were reduced in participants with P-tau levels >70 ng/l, regardless of Aβ_42_-status. This could also be interpreted as support for the hypothesis that in early stages the posterior subiculum is more sensitive to AD-pathology than the anterior part. A cutoff for P-tau of 52 ng/l is low. But also CN with P-tau >52 ng/l displayed a tendency to reduction in the volume of the posterior but not anterior left subiculum compared to CN with levels below that of cutoff (data not shown).

Together these data clearly show the advantage of considering the differences between the anterior vs. posterior part of single subfields when studying how pathological processes affect the HC. To our knowledge, this has not been considered in a systematic way in previous studies. One reason why this has received so little attention in MRI studies is probably that current subfield terminology does not subdivide single subfields in the anterior-posterior direction (Duvernoy and Bourgouin, 1998). However, knowledge about the functional specialization of anterior vs. posterior HC has improved during recent decades (reviewed in Fanselow and Dong, 2010; Strange et al., 2014). This is also paralleled by increasing knowledge about the cellular characteristics within specific HC subfields. Two studies on the Allen Brain Atlas^3^ of C57BI/6 mice found that the pyramidal neurons of CA1 and CA3 displayed a clear laminar specificity in spatially distinct molecular domains distributed along the anterior-posterior axis (Thompson et al., 2008; Dong et al., 2009). Genes that are specifically expressed in the CA1, CA2 or CA3 are also clearly segregated among the dorsal, intermediate and ventral segments of these subfields (Dong et al., 2009; Fanselow and Dong, 2010). In recent parcellation models for CA1 and CA3 these subfield are divided into CA1 and CA3 d (dorsal), CA1 and CA3 i (intermediate), and CA1 and CA3 v (ventral; Fanselow and Dong, 2010). While less studied, there is evidence from neuropathological studies suggesting that the structural and functional subdomains found in CA1 are also present in the subiculum (Risold and Swanson, 1996; Swanson, 2004). Altogether, many studies support a subdivision of the HC subfields along the anterior-posterior axis. Another reason why this has not been considered in currently used subfield segmentation approaches is probably that the boundaries between the dorsal, intermediate and posterior parts of HC are not as clearly defined as those between the different subfields (Dong et al., 2009). A distinct division based on connectivity or on function will probably never be possible as changes occur gradually along the axis of HC (Aggleton, 2012; Strange et al., 2014). A possible way of handling this is the approach taken in this study in which one HC subfield was divided into 10 equally long sections along the long axis of the HC.

While our plots indicate that a posterior > anterior atrophic gradient is present in subiculum bilaterally in participants with abnormal levels of CSF Aβ_42_ and P-tau, does this effect seem to be particularly pronounced on the left side (see Figures 2, 3, 7). We also found that the left posterior subiculum was affected earlier by elevated P-tau levels than the left anterior subiculum in participants with normal CSF Aβ_42_. This interaction was not significant on the right side. A left > right rate of atrophy has been found by Morra et al. (2009) in a large longitudinal study including 490 subjects. The authors propose that the right hemisphere may potentially lag behind the left in the very early stages of dementia. A left > right hippocampal volume loss has also been found in cross-sectional studies. Qiu et al. (2008) found that cognitively normal subjects with a Clinical Dementia Rating scale (CDR) of 0.5 (questionable AD) displayed atrophy of the left lateral hippocampal tail. We found atrophy in the left but not the right hippocampal body and tail in a group of patients with mild AD compared to controls subjects (Lindberg et al., 2012). Apostolova et al. (2010) investigated 169 amnestic MCI patients and found left > right atrophy of the hippocampal body and tail in participants who later converted to AD. They propose that this may be related to an ascertainment bias as standardized cognitive testing for patients suspected of having a dementia diagnosis is biased towards verbally mediated tasks which are more sensitive to left-side pathology. It is possible that our finding is partly driven by a selection bias as suggested in the study by Apostolova et al. (2010). However, CN subjects with low Aβ_42_-levels also have decreased volume of the total left (*F*_(1,283)_ = 4.52, *p* = 0.03) but not right (*F*_(1,277)_ = 1.19, *p* = 0.28) subiculum (Supplementary Figures S4A,B), which indicates that there may be an underlying biological mechanism that contributes to this asymmetry.

Another finding that may be related to a selection bias is that SCD patients without pathology also seem to have more atrophy of the posterior subiculum. Furthermore, in MCI patients with normal levels of Aβ_42_ we see an interaction between diagnosis (CN vs. MCI) and location (Supplementary Figure S2). We believe that this can be explained by a selection bias in which people with atrophy of the posterior hippocampal system are more likely to be diagnosed as preclinical AD than people with atrophy of the anterior system.

The most important limitation of this study is that only one subfield was analyzed. It would of course strengthen our argument if we could show a more significant anterior-posterior atrophic gradient in other subfields that have previously been shown to be affected in AD, including CA1. However, we do not consider the segmentations of subfields other than presubiculum reliable when using FS 5.3. Another limitation is that the hippocampal tail is excluded in FS 5.3 (and 6). It is possible that we would have fond an even stronger association between AD pathology and the posterior subiculum if we could have included measurements from the hippocampal tail.

It has been reported that FS 6 provides a better parcellation scheme than FS 5.3 which is more consistent with known histopathological anatomical borders of hippocampal subfields (Iglesias et al., 2015). However in addition to an improved parcellation schema FS6 is also parcellating the molecular cellular layer (Iglesias et al., 2015). But while the molecular layer is present in the different subparts of the subiculum, the parcellation scheme only provides the total volume of the molecular layer. This creates a difficult problem in terms of quality control. To visually inspect if the molecular layer is correctly segmented we need a MRI image with very high resolution. On regular structural MRI images we are basically forced to accept the segmentation provided by FS6 without any possibility to do quality control of the results. If the molecular layer had been subdivided so that we would know how much volume of this layer that belongs to each subdivision of the subiculum, we could ourselves determine whether to present the total volume of each subicular subdivision or the molecular and other cellular layer separately based on the resolution of the MRI-image in the study. In FS 5.3 we are still able to do a visual quality control of the subicular subfield which is the reason why this older version was used in this study.

One further limitation is that two results have a relatively weak *p*-value (≥0.045). We have emphasized this by denoting them as borderline significant. The interaction significant between high vs. low levels of Aβ_42_ and location for the left subiculum in SCD, as well as the interaction between diagnosis CN normal vs. SCD high levels of P-tau and location for the right subiculum thus needs to be validated in a replication study.

Finally it should be acknowledged that the hypothetical model that relates episodic memory particularly to the functionality of the posterior HC has been challenged in some recent articles (see Zeidman and Maguire, 2016). As presented, we found that the posterior part of the subiculum was generally more associated with performance on delayed recollection than the anterior part.

The main goal of the study was to investigate if we could confirm whether specific regions of a single subfield along the anterior-posterior axis of HC could be selectively vulnerable to pathology. We found strong support for this hypothesis. If the whole volume of the subiculum had been considered, no effect of CSF Aβ_42_ or P-tau would have been found in the SCD group. Low CSF Aβ_42_ was however associated with significant volume loss of the left posterior subiculum, while elevated P-tau was associated with volume loss in the right posterior subiculum.

In conclusion, we have shown that emerging early Aβ and tau pathology, predominantly affects the posterior subiculum which suggests that future segmentations approaches of HC subfields would benefit from considering volumetric differences along the anterior-posterior axis.

## Author Contributions

Study concept and design was formulated by OL. GM created the application for dividing the subicular subfield from Freesurfer into volumetric subsection. Acquisition, analysis or interpretation of data were done by OL, GM, ES, SP, L-OW, EW, OH.

## Conflict of Interest Statement

OH has acquired research support (for the institution) from Roche, GE Healthcare, Biogen, AVID Radiopharmaceuticals, Fujirebio, and Euroimmun. In the past 2 years, he has received consultancy/speaker fees (paid to the institution) from Lilly, Roche, and Fujirebio. The other authors declare that the research was conducted in the absence of any commercial or financial relationships that could be construed as a potential conflict of interest.

## Footnotes

^1^www.biofinder.se

^2^http://surfer.nmr.mgh.harvard.edu/

^3^www.brain-map.org
