# Supplementary material for: Atrophy of the Posterior Subiculum Is Associated with Memory Impairment, Tau- and Aβ Pathology in Non-demented Individuals
Source: Front Aging Neurosci. 2017 Sep 20;9:306. doi: 10.3389/fnagi.2017.00306 (PMC5611434; doi:10.3389/fnagi.2017.00306)
Supplement: Supplementary file 1 [file Presentation_1.pptx]

## Slide 1
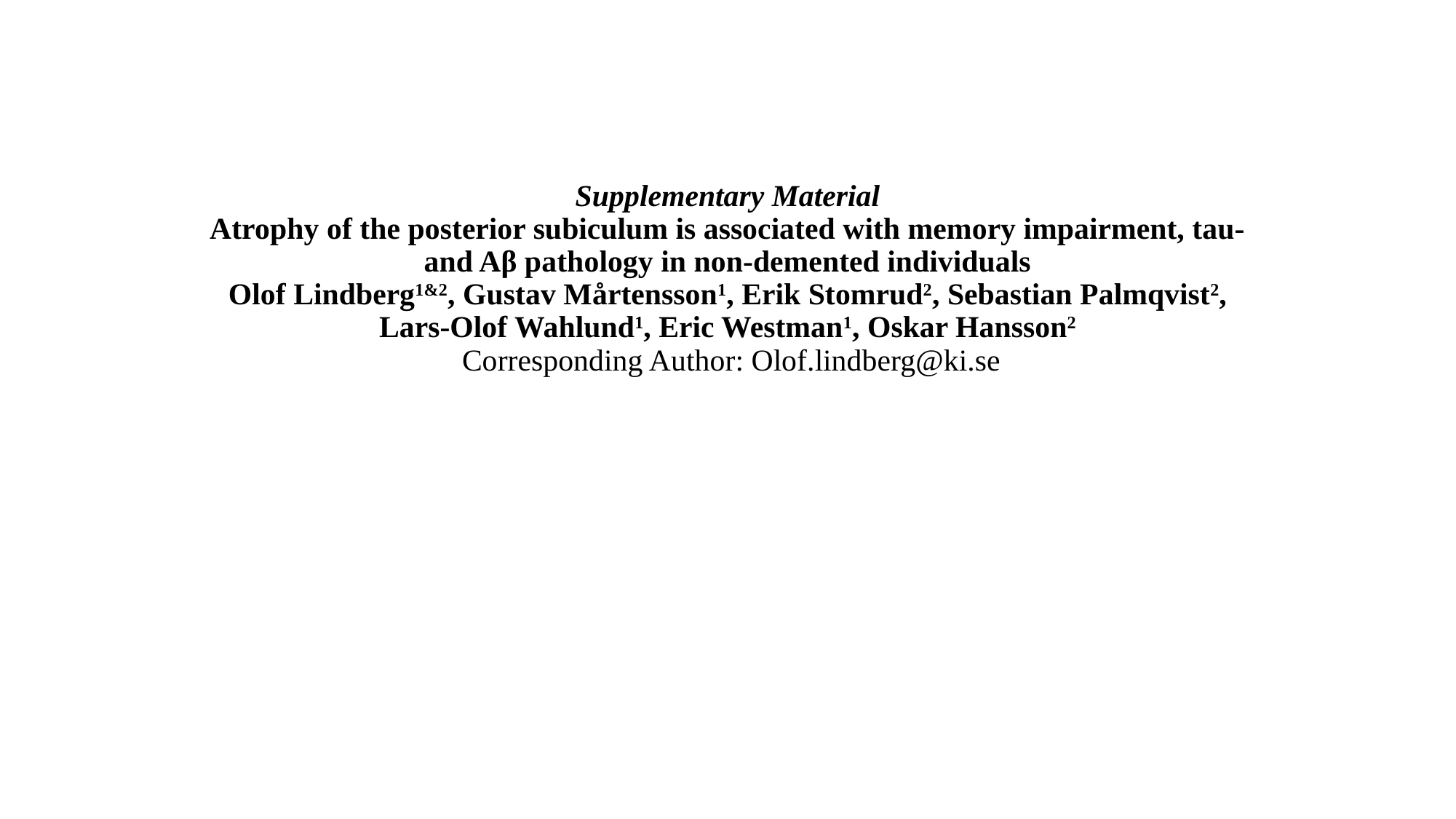

# Supplementary Material Atrophy of the posterior subiculum is associated with memory impairment, tau- and Aβ pathology in non-demented individuals Olof Lindberg1&2, Gustav Mårtensson1, Erik Stomrud2, Sebastian Palmqvist2, Lars-Olof Wahlund1, Eric Westman1, Oskar Hansson2 Corresponding Author: Olof.lindberg@ki.se

## Slide 2
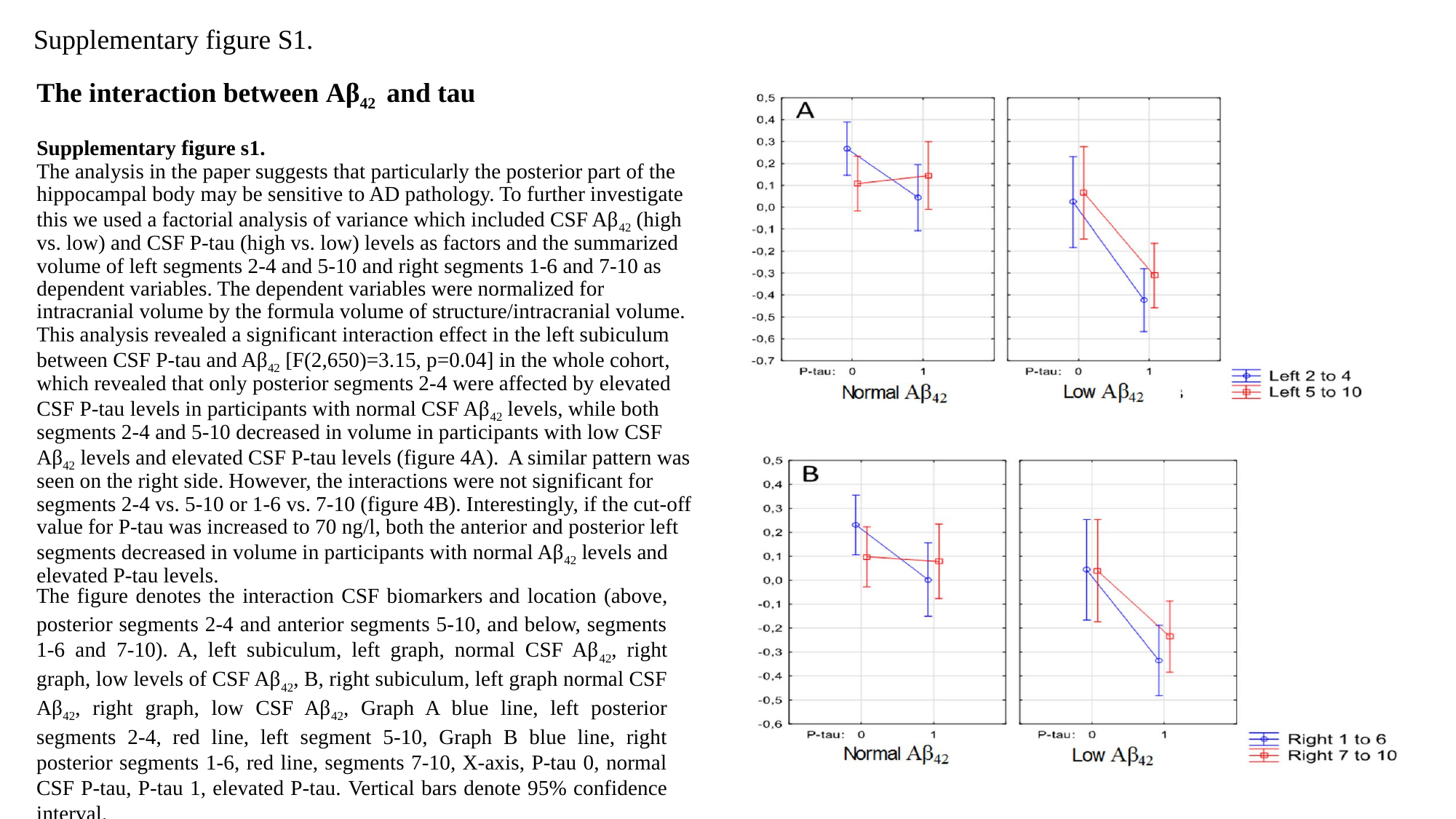

Supplementary figure S1.
The interaction between Aβ42 and tau
Supplementary figure s1.
The analysis in the paper suggests that particularly the posterior part of the hippocampal body may be sensitive to AD pathology. To further investigate this we used a factorial analysis of variance which included CSF Aβ42 (high vs. low) and CSF P-tau (high vs. low) levels as factors and the summarized volume of left segments 2-4 and 5-10 and right segments 1-6 and 7-10 as dependent variables. The dependent variables were normalized for intracranial volume by the formula volume of structure/intracranial volume. This analysis revealed a significant interaction effect in the left subiculum between CSF P-tau and Aβ42 [F(2,650)=3.15, p=0.04] in the whole cohort, which revealed that only posterior segments 2-4 were affected by elevated CSF P-tau levels in participants with normal CSF Aβ42 levels, while both segments 2-4 and 5-10 decreased in volume in participants with low CSF Aβ42 levels and elevated CSF P-tau levels (figure 4A). A similar pattern was seen on the right side. However, the interactions were not significant for segments 2-4 vs. 5-10 or 1-6 vs. 7-10 (figure 4B). Interestingly, if the cut-off value for P-tau was increased to 70 ng/l, both the anterior and posterior left segments decreased in volume in participants with normal Aβ42 levels and elevated P-tau levels.
The figure denotes the interaction CSF biomarkers and location (above, posterior segments 2-4 and anterior segments 5-10, and below, segments 1-6 and 7-10). A, left subiculum, left graph, normal CSF Aβ42, right graph, low levels of CSF Aβ42, B, right subiculum, left graph normal CSF Aβ42, right graph, low CSF Aβ42, Graph A blue line, left posterior segments 2-4, red line, left segment 5-10, Graph B blue line, right posterior segments 1-6, red line, segments 7-10, X-axis, P-tau 0, normal CSF P-tau, P-tau 1, elevated P-tau. Vertical bars denote 95% confidence interval.

## Slide 3
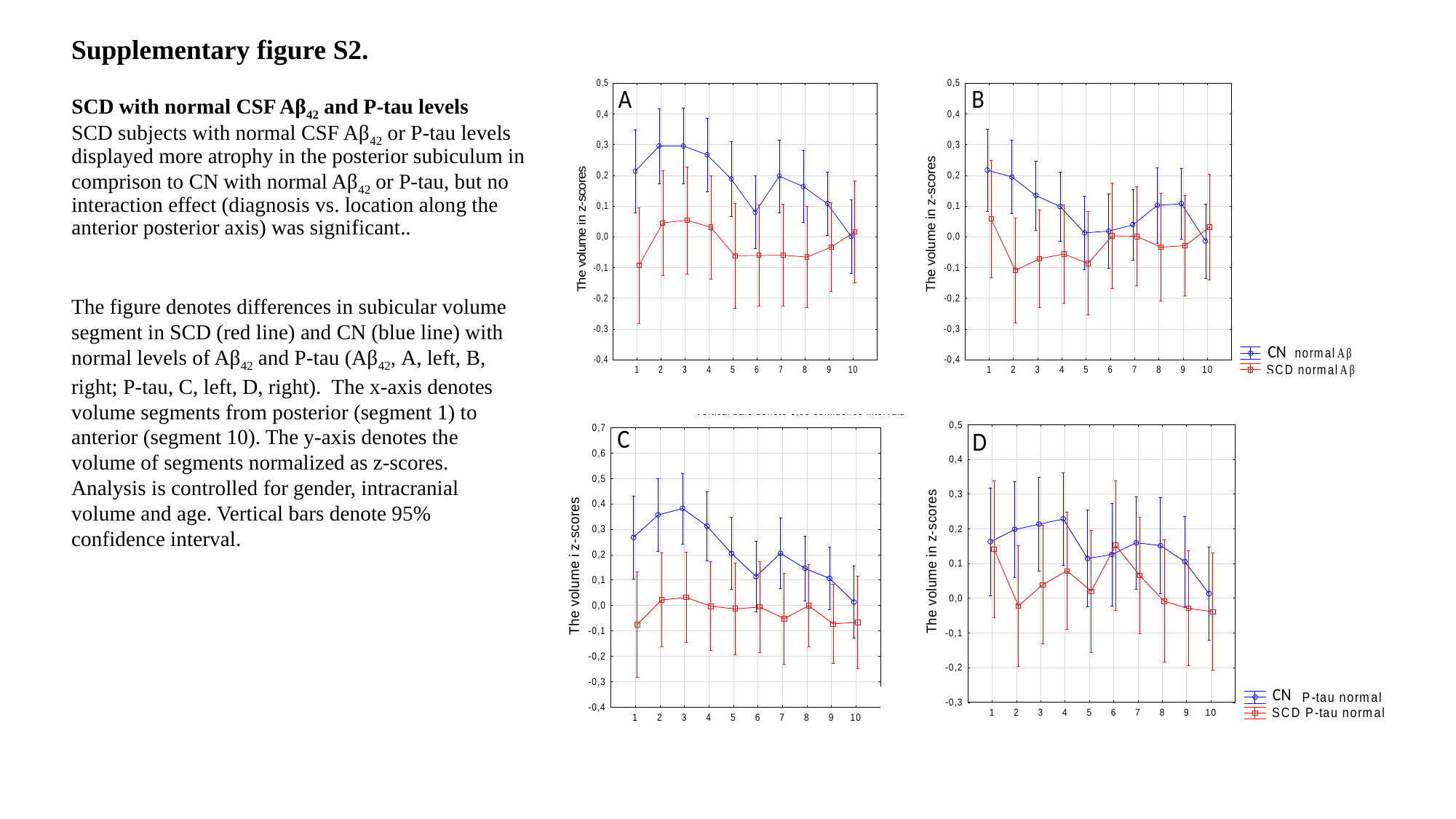

Supplementary figure S2.
# SCD with normal CSF Aβ42 and P-tau levels SCD subjects with normal CSF Aβ42 or P-tau levels displayed more atrophy in the posterior subiculum in comprison to CN with normal Aβ42 or P-tau, but no interaction effect (diagnosis vs. location along the anterior posterior axis) was significant..
The figure denotes differences in subicular volume segment in SCD (red line) and CN (blue line) with normal levels of Aβ42 and P-tau (Aβ42, A, left, B, right; P-tau, C, left, D, right). The x-axis denotes volume segments from posterior (segment 1) to anterior (segment 10). The y-axis denotes the volume of segments normalized as z-scores. Analysis is controlled for gender, intracranial volume and age. Vertical bars denote 95% confidence interval.

## Slide 4
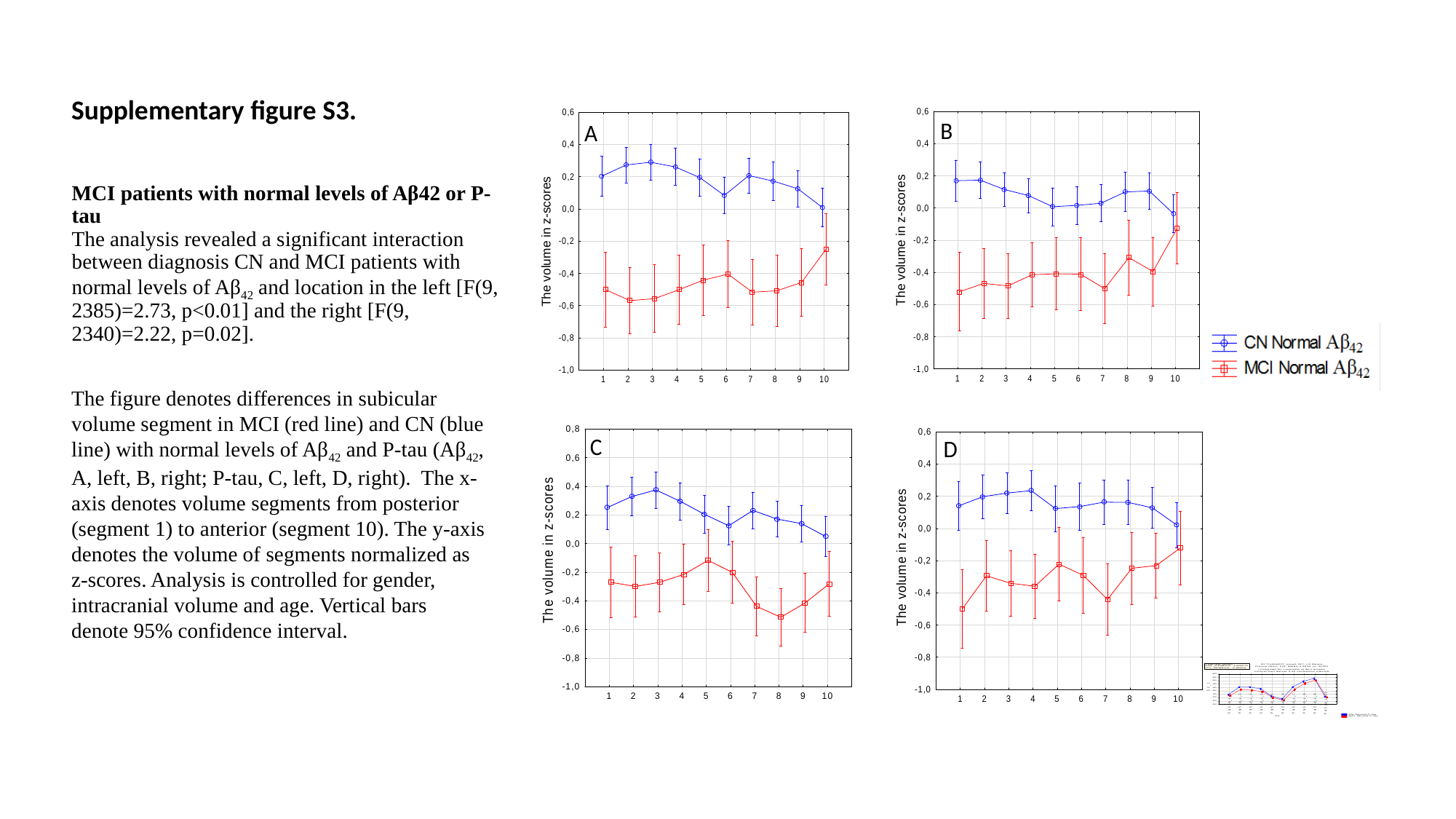

Supplementary figure S3.
# MCI patients with normal levels of Aβ42 or P-tau The analysis revealed a significant interaction between diagnosis CN and MCI patients with normal levels of Aβ42 and location in the left [F(9, 2385)=2.73, p<0.01] and the right [F(9, 2340)=2.22, p=0.02].
The figure denotes differences in subicular volume segment in MCI (red line) and CN (blue line) with normal levels of Aβ42 and P-tau (Aβ42, A, left, B, right; P-tau, C, left, D, right). The x-axis denotes volume segments from posterior (segment 1) to anterior (segment 10). The y-axis denotes the volume of segments normalized as z-scores. Analysis is controlled for gender, intracranial volume and age. Vertical bars denote 95% confidence interval.

## Slide 5
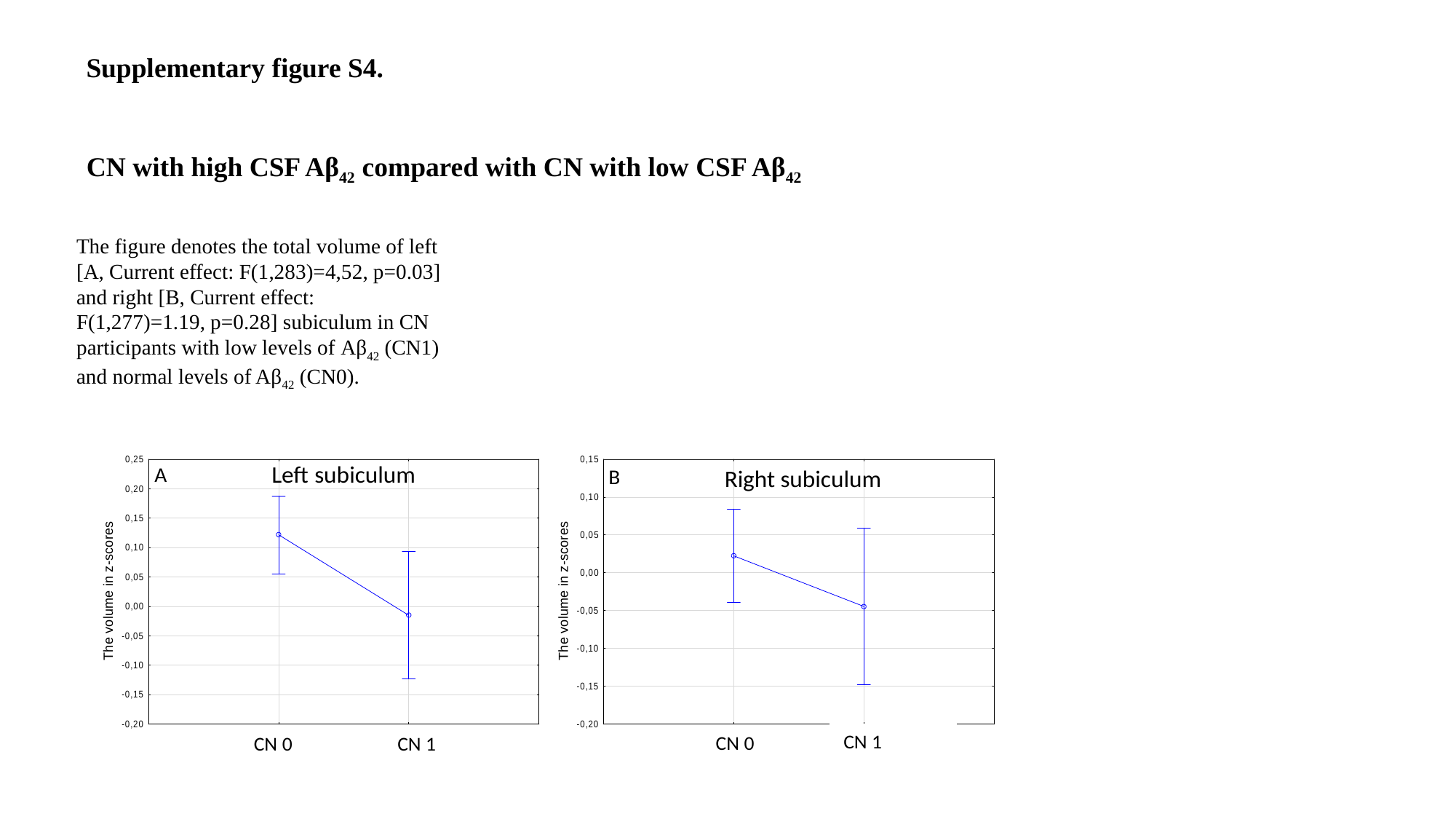

Supplementary figure S4.
# CN with high CSF Aβ42 compared with CN with low CSF Aβ42
The figure denotes the total volume of left [A, Current effect: F(1,283)=4,52, p=0.03] and right [B, Current effect: F(1,277)=1.19, p=0.28] subiculum in CN participants with low levels of Aβ42 (CN1) and normal levels of Aβ42 (CN0).
